# Interrelation among exercise training, cardiac hypertrophy, and tissue kallikrein-kinin system in athlete and non-athlete women

**DOI:** 10.34172/jcvtr.2022.28

**Published:** 2022-08-30

**Authors:** Behnam Heidari, Mohammad Reza Zolfaghari, Kamal Khademvatani, Amir Fattahi, Reza Zarezadeh

**Affiliations:** ^1^Department of Physical Education, Faculty of Sport Sciences, Urmia University, Urmia, Iran; ^2^Cardiology Department, School of Medicine, Urmia University of Medical Sciences, Urmia, Iran; ^3^Department of Reproductive Biology, Faculty of Advanced Medical Sciences, Tabriz University of Medical Sciences, Tabriz, Iran; ^4^Department of Biochemistry and Clinical Laboratories, Faculty of Medicine, Tabriz University of Medical Sciences, Tabriz, Iran

**Keywords:** Bradykinin, Cardiomegaly, Exercise, Kallikrein-Kinin System, Tissue Kallikreins

## Abstract

***Introduction:*** The tissue kallikrein-kinin system is an endogenous homeostatic pathway, which its stimulation is associated with cardioprotection. The present study aimed to determine the effect of exercise training on plasma tissue kallikrein (TK) and bradykinin (BK) and their association with cardiac hypertrophy.

***Methods:*** 22 non-athlete and 22 athlete women were exposed to acute (Bruce test) and chronic (12-week swimming training) exercises. 2D echocardiography was used to evaluate morphological and functional features of the heart. Plasma concentrations of TK and BK were quantified by ELISA.

***Results:*** Athletes had significantly higher values of left ventricle end-diastolic diameter index (LVEDDI) and left ventricle mass index (LVMI) than non-athletes. Exercise intervention affected echocardiographic features in neither of the study groups. Chronic exercise training notably increased plasma levels of TK and BK, which increase was more pronounced in the athletes. Plasma TK negatively correlated with LVEDDI (r=−0.64, *P*=0.036 and r=−0.58, *P*=0.027) and LVMI (r=−0.51, *P*=0.032 and r=−0.63, *P*=0.028) in the non-athlete and athlete groups. In opposition, there was a positive correlation between plasma TK and left ventricle ejection fraction in non-athletes (r=0.39, *P*=0.049) and athletes (r=0.53, *P*=0.019).

***Conclusion:*** The upregulation of the tissue kallikrein-kinin system may be a protective mechanism against excessive cardiac hypertrophy induced by chronic exercise training.

## Introduction

 As a consequence of functional overload, the heart undergoes an increase in size and mass, a phenomenon called cardiac hypertrophy. Functional overload typically results from a variety of physiological and pathological circumstances. Accordingly, cardiac enlargement is dichotomized into physiological and pathological types. Physiological cardiac hypertrophy occurs in response to exercise training and is accompanied by normal heart anatomy, preserved or even improved myocardial contractility, and usually regresses with detraining. In contrast, pathological cardiac hypertrophy occurs as a result of abnormalities such as hypertension, ischemia, or diabetes and is accompanied by cardiomyocyte apoptosis, fibrotic response, diminished neovascularization, and cardiac dysfunction, raising the probability of heart failure. In addition to structural, histological, and functional aspects, molecular mechanisms underlying these two types of hypertrophies are distinct from each other.^1^

 Cardiac hypertrophy is a divergent phenomenon. It exhibits specific architectural and functional features relying on the causative mechanism.^2^ Globally, cardiac hypertrophy is divided into two major categories: concentric and eccentric.^3^ Concentric geometry refers to hypertrophies without ventricular dilation and results from pressure overload in the left ventricle. On the other side, eccentric architecture represents patterns with a dilated chamber as a consequence of pregnancy, repetitive exercise training, and mitral regurgitation.^4^ Regarding its validity, noninvasiveness, and ease of access, echocardiography is the method of choice for the detection of cardiac hypertrophy in the clinical setting.^2,3^ From the viewpoint of echocardiographic parameters, concentric hypertrophy is manifested through a normal ventricular end-diastolic diameter (LVEDD) concomitant with an increase in the left ventricle mass (LVM). On the contrary, eccentric hypertrophy is recognized by an increase in both LVEDD and LVM.^4^

 The kallikrein-kinin system is an endogenous homeostatic pathway, which its stimulation leads to the liberation of vasoactive kinins. This complicated system consists of the precursors of kinins, namely kininogens, along with plasma and tissue kallikreins, which act on low- and high-molecular-weight kininogens to produce and release kinins. Tissue kallikrein (TK) is a serine protease that is encoded by the KLK1 gene and proteolytically processes kininogens to liberate vasoactive peptides bradykinin (BK) and lys-bradykinin.^5,6^ There is ample evidence that TK has antihypertrophic and cardioprotective effects against numerous pathological conditions. For example, hypertrophic transgenic rats overexpressing human TK displayed attenuated myocardial size and weight along with enhanced cardiac function.^7^ Similarly, human TK-enriched mesenchymal stem cells alleviated cardiac damage and loss of cardiomyocytes and enhanced angiogenesis after myocardial infarction, which altogether resulted in the restoration of cardiac function.^8^ Of note, evidence on kinin B_1_ and B_2_ receptor-null mice indicated that the beneficial effects of the kallikrein-kinin system on cardiac damage caused by ischemia/reperfusion are mediated by B_2_ receptors rather than B_1_ ones.^9^

 In humans, a polymorphic variant of kinin B_2_ receptor associated with lower gene expression was found to be concomitant with greater cardiac hypertrophy following chronic exercise training.^10^ Furthermore, it has been demonstrated that mice with B_2_ receptor deficiency developed pathologic hypertrophy in the left ventricle in response to chronic physical training, whereas their normal counterparts underwent physiological hypertrophy, suggesting an active role of kinin B_2_ receptor in the development of physiological hypertrophy.^11^ According to a more recent study, regular physical exercises increased the myocardial expression of TK and kinin B_2_ receptors in rat models.^12^ However, the association of upstream kallikrein-kinin elements, i.e. TK and BK, with exercise-induced cardiac hypertrophy as well as with exercise training is not fully understood. Here, we firstly aimed to determine the effect of acute and chronic exercise training on plasma levels of TK and BK in a population of athlete and non-athlete women. Secondly, we aimed to evaluate the association of plasma TK and BK with the morphological and functional features of cardiac muscle.

## Materials and methods

###  Study population

 The study population consisted of 22 non-athlete women with a sedentary lifestyle along with 22 athlete women with regular physical training (at least 5 workout sessions per week and each session for 2 h). The sample size was calculated using PS Power and Sample Size Calculations software (version 3.0) by inputting 0.05 for the type 1 error, 0.8 for power, and 1 for the ratio of athletes to non-athletes as well as the values of difference in group means and within-group standard deviation, which were obtained from previous studies. It should be noted that 10 people out of each group were subjects of our previous study.^13^ All participants were chosen from premenopausal women with a mean age of 30 ± 10. Individuals with a history of drug use in 3 weeks prior to the study, cigarette smoking, menopause, and cardiovascular, renal, hepatic, and endocrine diseases were excluded from the study. The subjects were ordered to follow a standard dietary regimen and did not receive any nutritional supplements. The inclusion of all women in the study was accomplished with their written informed consent. Urmia University approved this study in accordance with the Declaration of Helsinki (Code: 68/459).

###  Exercise intervention and specimen collection

 The study population was subjected to acute and chronic physical training, respectively. For acute exercise intervention, the Bruce’s treadmill protocol was carried out on the participants. The Bruce test is one of the most widespread exercise examinations utilized in clinical practice and needs a significant amount of energy use.^14^ The test began with a low workload as a warm-up and continued with five subsequent progressive rounds in terms of speed and slope as follows: round 1, 2.7 km/h and 10% slope; round 2, 4 km/h and 12% slope; round 3, 5.5 km/h and 14% slope; round 4, 6.8 km/h and 16% slope; round 5, 8 km/h and 18% slope, which each round lasted 3 min. The test ended by reaching 85% of each participant’s maximum heart rate.^15^ Before and after this intensive exercise, blood specimens were taken in anticoagulant-containing tubes and were stored at −80°C for future analyses. For chronic exercise intervention, participants were subjected to aerobic swimming training 3 sessions per week, and overall for 12 weeks. These training started with low intensity (60% of maximum heart rate) and at 30 min intervals in each session, but following the subjects (particularly non-exercising ones) became physically stronger, the interval and intensity of training were raised to 40−60 min and 70−80% of maximum heart rate, respectively. To rule out the interfering impact of the acute dimension of training, blood sampling was performed on the next day of the last exercise session. With the purpose of evaluating the long-term influences of physical training, 3 days (72 hours) after the latter sampling, the subjects also underwent venipuncture and blood withdrawal, meanwhile; they took no exercise after the last swimming session. Sampling time points are chronologically summarized in Table 1.

**Table 1:** Chronological sampling time points

**Sampling time point**	**Explanation**
T1	Prior to acute and chronic exercise
T2	After acute exercise
T3	1 day (24 hours) after the end of chronic exercise period
T4	4 day (96 hours) after the end of chronic exercise

###  Echocardiography

 All participants were subjected to electrocardiographic examination before and after the exercise intervention to measure heart rate, septal thickness, posterior wall thickness (PWT), left ventricular ejection fraction (LVEF), left ventricular end-diastolic diameter index (LVEDDI), left ventricular mass index (LVMI), end-systolic stress (ESS), and relative wall thickness (RWT) using the apical window. LVEF was calculated based on Simpson’s method with two apical views.^16^ LVMI was calculated according to the Devereux formula^17^ and was indexed for body surface area. RWT was obtained by multiplying the PWT to the left ventricular internal diastolic dimension (LVIDD) ratio by 2.^18^ In accordance with the method introduced by Reichek et al^19^, ESS was also assessed based on the systolic blood pressure.

###  Measurement of plasma parameters

 The concentrations of TK and BK in plasma were quantified using commercial enzyme-linked immunosorbent assay (ELISA) kits (CUSABIO, China). The limit of detection was 0.195 ng/ml for TK and 0.078 pg/mL for BK. Additionally, intra-assay and inter-assay coefficients of variation (CV) were < 8% and < 10% for both TK and BK, respectively.

###  Statistical analysis

 The One-sample Kolmogorov-Smirnov test was carried out to confirm the normality of data distributions. The value of variables was presented as mean ± SD. Variables were compared between non-athlete and athlete groups by the independent-samples t-test. The paired-samples t-test was used to evaluate data before and after the exercise training course. The homoscedasticity of variances was confirmed by Leven’s test and two-way ANOVA was performed to identify differences between the groups and among the sampling days. The possible correlations between variables were investigated by the Pearson correlation test. IBM SPSS software (version 19.0, SPSS Inc., Chicago, IL, USA) was utilized to conduct all statistical analyses. *P *values < 0.05 were considered as statistically significant.

## Results

###  Demographic and echocardiographic features of the subjects

 Athlete and non-athlete (control) groups did not significantly differ in terms of age (30.51 ± 6.40 vs. 32.48 ± 6.94 years, *P* > 0.05) and BMI (25.1 ± 4.8 vs. 22.9 ± 3.4 kg/m^2^, *P* > 0.05). According to the echocardiographic findings (Table 2), athletes had statistically higher baseline values of LVEDDI and LVMI than the non-athlete population (*P* < 0.05). After 12-week water aerobic exercises, while LVMI remained significantly higher in the athlete group compared to the control group (*P* < 0.05), LVEDDI reached levels that were similar between the groups (*P* > 0.05). Furthermore, no statistical difference in echocardiographic features was noted in either of the groups before and after the exercise intervention (*P* > 0.05).

**Table 2:** Echocardiographic features before and after a 12-week aerobic ‎exercise training in non-athlete and athlete women

**Echocardiographic features**	**Non-athlete** **(n=22)**		**Athlete** **(n=22)**	
	**Before**	**After**	**Before**	**After**
Septal thickness (mm)	8.4 ± 2.1	8.3 ± 1.7	8.8 ± 1.4	8.7 ± 2.3
Posterior wall thickness (mm)	8.7 ± 0.9	8.8 ± 1.5	9.1 ± 1.6	8.9 ± 2.2
RWT (%)	40.1 ± 8.6	41.9 ± 9.1	44.1 ± 9.7	42.7 ± 6.9
LVEDDI (mm/m^2^)	25.3 ± 5.1	26.2 ± 4.9	28.5 ± 5.1^*^	27.9 ± 5.3
LVMI (g/m^2^)	78.2 ± 8.1	77.2 ± 6.6	84.3 ± 7.8^*^	85.1 ± 9.0^*^
ESS (kdyne/cm^2^)	68 ± 6	70 ± 8	72 ± 7	71 ± 9
LVEF (%)	62.5 ± 10.1	64.0 ± 11.5	64.9 ± 8.3	65.7 ± 9.4

Abbreviations: ESS, end-systolic stress; LVEDDI, left ventricular end-diastolic diameter index; LVEF, left ventricular ejection fraction; LVMI, left ventricular mass index; RWT, Relative wall thickness * Significant difference (*P* < 0.05) between the non-athlete and athlete groups.

###  Plasma parameters in relation to exercise training

**Figure 1:**
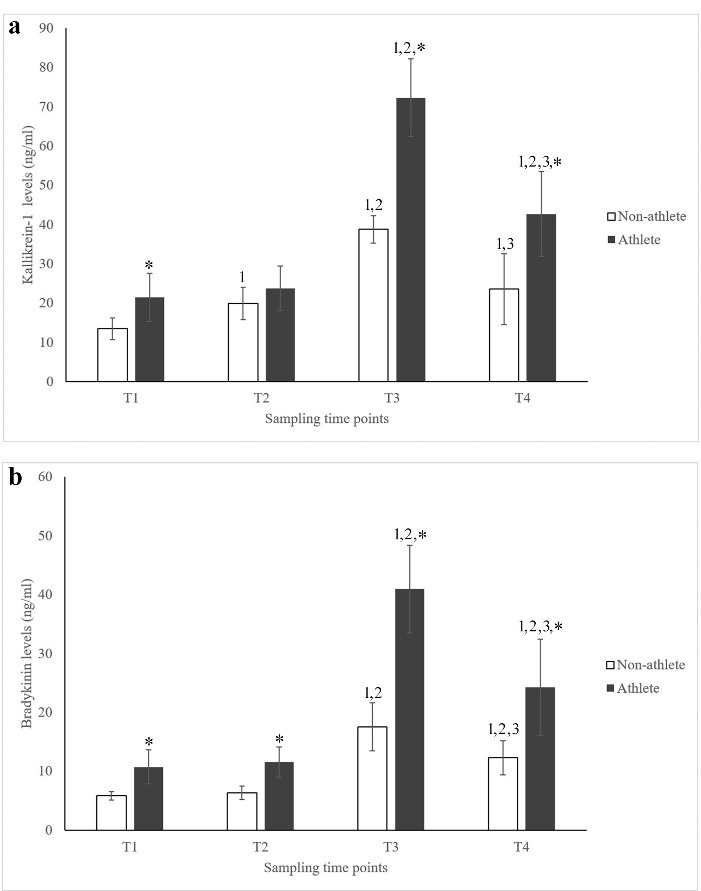
Plasma levels of (a) tissue kallikrein and (b) bradykinin following acute and chronic exercise training in non-athlete (n = 22) and athlete (n = 22) women. Data are shown as mean ± S.D. The homoscedasticity of variances was confirmed by Levene’s test and two-way ANOVA was performed to identify differences between the groups and among the sampling time points. ^1,2,3^ Significant difference (*P* < 0.05) in comparison with T1, T2, and T3 in the same group, respectively. * Significant difference in comparison with the non-athlete group at the same time point.

 Plasma levels of TK in the study groups at different time points, namely before exercise (T1), immediately after acute exercise (T2), and 24 h and 72 h after chronic exercise (T3 and T4, respectively), are illustrated in Figure 1a. Accordingly, acute training led to a significant increase in the plasma concentrations of TK in the non-athlete subjects (*P* < 0.05). Similarly, the plasma levels of TK in the control group dramatically elevated 24 h after chronic exercise compared to baseline and after-acute training time points (*P* < 0.05). Although plasma amounts of TK statistically decreased in the non-athlete individuals 4 days after chronic exercise training, TK plasma concentrations were still significantly higher than those of the T1 time point (*p* < 0.05). Among the athlete subjects, while acute exercise did not make a statistical alteration in the plasma concentrations of TK (*P* > 0.05), long-term training dramatically increased its plasma levels at the T3 time point (*P* < 0.05). Similar to the control group, although plasma amounts of TK significantly decreased in the athlete group 4 days after chronic exercise, TK plasma concentrations were still significantly higher than those of T1 and T2 time points (*P* < 0.05). Moreover, comparing the study groups demonstrated that athletes had significantly higher concentrations of plasma TK in all sampling time points except for T2 (*P*< 0.05).

 Plasma levels of BK in the study groups at different time points are illustrated in Figure 1b. Accordingly, the plasma concentrations of BK were not significantly affected in both groups following acute training (*P* > 0.05). Conversely, plasma levels of BK dramatically elevated among the athlete and non-athlete subjects 24 h after chronic exercise compared to baseline and after-acute training time points (*P*< 0.05). Although 4 days after long-term training, the plasma amounts of BK statistically decreased in both groups, BK plasma concentrations were still higher than those of T1 and T2 time points (*P* < 0.05). Furthermore, athlete individuals displayed significantly elevated plasma levels of BK compared to their non-athlete counterparts (*P* < 0.05).

###  Correlation between echocardiographic features and plasma parameters

 The Pearson correlation analysis was performed to disclose probable correlations between echocardiographic features and plasma levels of TK and BK in the subjects (Table 3). Accordingly, plasma TK negatively correlated with LVEDDI in both non-athlete (r = −0.64, *P* = 0.036) and athlete (r = −0.58, *P* = 0.027) groups. Similarly, there were significant inverse correlations between plasma TK and LVMI in the control and athlete groups (r = −0.51, *P* = 0.032 and r = −0.63, *P* = 0.028, respectively). Moreover, plasma TK displayed significantly positive correlations with LVEF in both non-athlete (r = 0.39, *P* = 0.049) and athlete (r = 0.53, *P* = 0.019) groups. In the case of BK, except for a significant negative correlation with LVMI (r = −0.46, *P* = 0.039) in the control group, no other statistically meaningful correlation was noted. It is also worthy of mention that the plasma levels of TK and BK positively correlated with each other in both the control (r = 0.709, *P*= 0.021) and athlete (r = 0.744, *P* = 0.014) groups.

**Table 3:** Correlations of plasma levels of tissue kallikrein and bradykinin with echocardiographic features in non-athlete and athlete women

**Echocardiographic features**	**Tissue kallikrein**				**Bradykinin**			
	**Non-athlete** **(n=22)**		**Athlete** **(n=22)**		**Non-athlete** **(n=22)**		**Athlete** **(n=22)**	
	**r**	***p***	**r**	***p***	**r**	***p***	**r**	***p***
Septal thickness (mm)	0.20	0.371	0.09	0.803	0.24	0.390	-0.15	0.544
Posterior wall thickness (mm)	0.10	0.742	0.30	0.285	0.09	0.820	-0.26	0.184
RWT (%)	0.34	0.072	0.11	0.682	0.16	0.803	-0.08	0.793
LVEDDI (mm/m^2^)	**-0.64**	**0.036**	**-0.58**	**0.027**	-0.25	0.211	-0.19	0.361
LVMI (g/m^2^)	**-0.51**	**0.032**	**-0.63**	**0.028**	**-0.46**	**0.039**	-0.22	0.134
ESS (kdyne/cm^2^)	-0.27	0.621	-0.38	0.382	-0.19	0.761	-0.30	0.506
LVEF (%)	**0.39**	**0.049**	**0.53**	**0.019**	0.22	0.270	0.31	0.163

Abbreviations: ESS, end-systolic stress; LVEDDI, left ventricular end-diastolic diameter index; LVEF, left ventricular ejection fraction; LVMI, left ventricular mass index; RWT, Relative wall thickness r values are Pearson’s correlation coefficients. Significant correlations are presented in bold.

## Discussion

 As a consequence of regular and heavy exercise training in athletes, the heart undergoes an enlargement in size, and its functional competence increases, the situation which is recognized as the athlete’s heart. These morphological and functional changes generally depend on a variety of factors such as the nature and intensity of exercise. According to a classical hypothesis in this respect, endurance sports trigger eccentric hypertrophy, whereas strength exercises give rise to concentric hypertrophy.^20^ In support of this hypothesis, Doronina et al^21^ found that while water polo training triggered volume enlargement and eccentric hypertrophy in the left ventricle among a cohort of female players, gentle concentric hypertrophy occurred in female bodybuilders. Consistent with the previous findings, we observed significantly higher values of left ventricle mass and diastolic volume in athlete individuals compared to non-athlete controls, indicating a physiological eccentric left ventricle hypertrophy in athletes. Despite this hypertrophic phenotype in athletes, a 12-week swimming exercise intervention led to no significant change in cardiac size and morphology in our non-athlete cohort. Similarly, untrained women within an age range similar to our population displayed no significant enhancement of cardiac size and function after three sessions per week of indoor cycling for 12 weeks.^22^ By contrast, a recent meta-analysis found that endurance exercise training is accompanied by the augmentation of left ventricle mass and diastolic volume (i.e. eccentric hypertrophy) in healthy inactive women.^23^ However, and concerning the substantial effect of exercise dose, consisting of its intensity, frequency, and duration, on training-induced cardiac adaptation,^22^ it should be noted that the duration of exercise interventions in most studies included in the latter meta-analysis was much longer than that of our study. Interestingly, the same meta-analysis also did not find a ventricular mass increase in female subjects after endurance training, when only studies that used echocardiography to evaluate cardiac phenotype were considered.^23^

 It has been revealed that regular physical exercise stimulates the kallikrein-kinin system in the myocardium of laboratory rat models. In detail, the expression of TK at both transcriptional and translational levels, as well as the mRNA expression of kinin B_2_ receptor, increased in the myocardial tissue of animals after a 13-week running exercise intervention.^12^ In agreement with these observations, we found a marked increase in the plasma levels of TK and BK following 12-week chronic swimming training in both control and athlete groups. Furthermore, and despite a marked reduction in plasma values of TK and BK during the post-exercise recovery period, they still remained at significantly higher levels compared to their concentrations at baseline and after acute exercise. These findings suggest that repetitive exercise training, but not intermittent training, gives rise to long-term activation of the kallikrein-kinin system by inducing a stable increase in the production of TK and BK. In support of this, we found significantly elevated amounts of TK and BK in the plasma of athlete individuals compared to their control counterparts. Moreover, it can be inferred from this observation that the plasma levels of TK and BK might be correlated with the duration of exercise training. In other words, the history of training could be a determining factor in the activation degree of the tissue kallikrein-kinin system. To the best of our knowledge, the present study is the first that has provided such data regarding the differential effect of acute and chronic exercise training on the plasma levels of TK and BK in sedentary and athlete subjects. However, the mechanism responsible for the release and increase of TK and BK following exercise training remained to be elucidated.

 There are several lines of evidence in animal models that TK exerts antihypertrophic and protective effects on the myocardium. For example, the delivery and expression of the human TK gene in the cardiac tissue markedly mitigated heart enlargement and dysfunction in rats exposed to pressure overload.^7^ Furthermore, Gao et al^8^ showed that human TK-transfected mesenchymal stem cells provided cardioprotection against myocardial infarction-induced hypertrophy and fibrosis mainly through the ability of TK to diminish ischemia-triggered cardiomyocyte apoptosis, leukocytes recruitment, and proinflammatory mediators’ expression. In accordance, our results demonstrated robust negative and positive correlations of plasma TK levels with size and contraction force of the left ventricle, respectively, implicating that elevated circulating concentrations of TK are associated with alleviated myocardial hypertrophy and improved cardiac function following exercise training. In opposition, the sole study linking TK to cardiovascular health in humans noticed a direct association between the plasma levels of TK and the risk of coronary artery disease.^24^ Paradoxically, the authors observed that plasma TK values were inversely correlated with the severity of coronary artery disease. For this reason, they suggested that elevation of plasma TK is a compensatory and protective response to atherosclerosis and thus high circulating TK content is a hallmark of functional impairment instead of a risk factor for coronary artery disease.

 The heart, and especially the left ventricle, undergoes an increase in mass and diameter during aging, a process that ultimately leads to cardiac hypertrophy, fibrosis, and dysfunction. The results of a study by Hu et al^25^ revealed that overexpression of human TK in the myocardium of aging rats prevented cardiomyocyte augmentation and consequently diminished morphological remodeling of the heart. On the other hand, evidence from studies on animal models suggests that physical training can counteract aging-induced cardiac alterations resulting in heart failure. Moreover, permanently exercised athletic elderlies have displayed lower age-associated changes in cardiac muscle, as compared to inactive older individuals.^26^ Interestingly, we noted that chronic exercise training increased plasma TK levels and athletes had markedly elevated plasma TK in comparison with sedentary subjects. Altogether, these observations proposed that the induction of TK expression and release may be an underlying mechanism, by which regular physical activity protects the heart against aging hallmarks.

 TK exerts its biological functions predominantly through the generation of kinin peptides and the subsequent activation of the kinin B_2_ receptor.^5^ In humans, diminished gene expression B_2_ receptor was found to be associated with pronounced left ventricular hypertrophy after a 10-week physical training course.^10^ According to Batista et al chronic swimming training led to cardiomyocyte enlargement and ventricular hypertrophy in both B_2_ receptor-ablated and wild-type mice. However, the former animals not only displayed more pronounced hypertrophy than the latter ones but also exhibited collagen accumulation and ventricular thickness, both of which are the hallmarks of pathologic hypertrophy.^11^ These findings suggest that the activation of the kinin B_2_ receptor is important for the development of physiologic hypertrophy rather than pathologic one in response to exercise training. Despite the observation of a negative correlation between left ventricular hypertrophy and plasma TK levels, we did not obtain such an association between left ventricular hypertrophy and plasma BK levels. A possible explanation for this inconsistency can be that the antihypertrophic effects of TK on the myocardium are not necessarily mediated by BK and alternative mechanisms are involved in this process. Interestingly, there is evidence for the ability of TK to afford cardioprotection by the direct activation of kinin B_2_ receptor in rats with kininogen deletion and thus kinin deficiency.^5^

 The results of *in vitro* and *in vivo* studies have reported molecular mechanisms by which TK protects the heart from hypoxia/reoxygenation damage. Accordingly, the stimulation of the kinin B_2_ receptor by TK/kinins activates AKT protein kinase, which in turn phosphorylates and thereby inhibits proapoptotic effects of GSK-3β and Bad, leading to the promotion of cardiomyocyte survival. Furthermore, the activation of the kinin B_2_ receptor by the tissue kallikrein-kinin system increases the production of nitric oxide (NO) through the induction of endothelial NO synthase. NO inhibits oxidative stress, JNK/p38MAPK, TGF-β1/Smad2, and NF-κB signaling pathways, which suppresses cardiac remodeling, fibrosis, and inflammation. A part of the cardioprotective effects of TK/kinins is due to the enhancement of angiogenesis as a result of GSK-3β inhibition *via* AKT phosphorylation, which elevates the intracellular levels of β-catenin and consequently upregulates the expression of vascular endothelial growth factor (VEGF) and VEGF receptor in endothelial cells.^5,27,28^ Although there is no mechanistic information about the action of the tissue kallikrein-kinin system on the cardiac muscle following exercise training, it can be hypothesized based on the evidence derived from ischemia/reperfusion models that upregulation of TK in response to exercise-induced overload stimulates the kinin B_2_ receptor, which activates AKT and increases NO formation, leading to the inactivation of GSK-3β, JNK/p38MAPK, TGF-β1/Smad2, and NF-κB signaling pathways with concomitant upregulation of VEGF. These molecular events in turn result in the decrement of oxidative stress, fibrosis, and inflammation along with the increment of neovascularization, which ultimately protects the heart from dramatic structural changes and hypertrophy.

 Some limitations of the present study need to be mentioned. Firstly, there might be differences in the heart’s morphological and functional parameters between the groups or before and after exercise intervention which 2D echocardiography was unable to detect, whereas sophisticated imaging modalities such as 3D echocardiography and cardiac MRI could disclose them. Secondly, the confounding contribution of plasma kallikrein activity toward plasma BK content was disregarded in the study. These shortcomings need to be addressed by future prospective studies on the association of exercise-induced cardiac hypertrophy with plasma TK and BK.

## Conclusion

 Our findings provided the first demonstration that the tissue kallikrein-kinin system is activated responding to chronic exercise training in both sedentary and athlete women. Moreover, plasma TK indicated an inverse association with post-exercise left ventricle hypertrophy, suggesting that TK upregulation is a protective mechanism against excessive cardiac hypertrophy induced by chronic exercise training.

## Acknowledgments

 The authors profoundly appreciate all the women who participated in the study as well as the medical personnel of Seyed-al-Shohada Cardiac Specialized Hospital for data collection.

## Funding

 This study was not supported by any grant funding.

## Ethical approval

 Urmia University approved this study in accordance with the Declaration of Helsinki (Code: 68/459).

## Competing interest

 The authors declare that they have no conflict of interest.
